# The hOGG1 Ser326Cys Gene Polymorphism and the Risk of Coronary Ectasia in the Chinese Population

**DOI:** 10.3390/ijms15011671

**Published:** 2014-01-22

**Authors:** Po-Chao Hsu, Chiao-Ling Wang, Ho-Ming Su, Suh-Hang Juo, Tsung-Hsien Lin, Wen-Chol Voon, Shyi-Jang Shin, Wen-Ter Lai, Sheng-Hsiung Sheu

**Affiliations:** 1 Division of Cardiology, Department of Internal Medicine, Kaohsiung Medical University Hospital, Kaohsiung 80708, Taiwan; E-Mail: pochao.hsu@gmail.com; 2 Graduate Institute of Medicine, College of Medicine, Kaohsiung Medical University, Kaohsiung 80708, Taiwan; E-Mail: bizibear@pchome.com.tw; 3 Department of Internal Medicine, Faculty of Medicine, Kaohsiung Medical University, Kaohsiung 80708, Taiwan; E-Mails: cobeshm@seed.net.tw (H.-M.S.); d750078@kmu.edu.tw (W.-C.V.); wtlai@cc.kmu.edu.tw (W.-T.L.); sheush@cc.kmu.edu.tw (S.-H.S.); 4 Department of Medical Research, Kaohsiung Medical University Hospital, Kaohsiung 80708, Taiwan; E-Mail: hjuo@kmu.edu.tw; 5 Medical Genetics, Kaohsiung Medical University, Kaohsiung 80708, Taiwan; 6 Center of Excellence for Environmental Medicine, Kaohsiung Medical University, Kaohsiung 80708, Taiwan; 7 Division of Endocrinology, Department of Internal Medicine, Kaohsiung Medical University Hospital, Kaohsiung 80708, Taiwan; E-Mail: sjshin@cc.kmu.edu.tw

**Keywords:** coronary ectasia, 8-oxoguanine DNA glycosylase, polymorphism

## Abstract

Oxidative stress (OS) is related to vascular inflammation possibly, contributing to the development of coronary ectasia (CE). Base excision repair (BER) and nucleotide excision repair are the main DNA repair pathways that can help to remove 8-hydroxydeoxyguanine (8-OHdG), a marker of OS. Human 8-oxoguanine DNA glycosylase 1 (hOGG1) is a key enzyme of the BER pathway and catalyzes the removal of 8-OHdG. The aim of our study was to investigate the association between hOGG1 Ser326Cys gene polymorphism and CE in a Chinese population. Five-hundred forty-seven patients who underwent diagnostic coronary angiography in a tertiary medical center were recruited. The angiographic definition of CE is the diameter of the ectatic segment being more than 1.5 times larger compared with an adjacent healthy reference segment. The gene polymorphisms were analyzed by polymerase chain reaction. The urine 8OHdG concentration was measured using a commercial ELISA kit. The distribution of hOGG1 Ser326Cys genotypes was significantly different between CE and non-CE groups (*p* = 0.033). The odds ratio of CE development for the Ser to the Cys variant was 1.55 (95% confidence interval (CI), 1.04–2.31, *p* = 0.033). Both univariate and logistic regression analysis showed a significant association of hOGG1 Ser326Cys polymorphism in the dominant model with CE development (*p* = 0.009 and 0.011, respectively). Urine 8-OHdG levels were significantly higher in subjects carrying the hOGG1 Ser variant than in those with the Cys/Cys genotype (*p* < 0.03). In conclusion, our study suggests that the hOGG1 Ser326Cys gene variant might play a role in susceptibility to the development of CE.

## 1. Introduction

Coronary ectasia (CE) is an uncommon disease, and its incidence is usually between 0.3% and 5% in different studies [1–5]. It has been defined as the diameter of the ectatic segment being more than 1.5 times larger compared with an adjacent healthy reference segment [2]. Most cases of CE are considered as a variant of coronary artery disease (CAD) [6]. Although the pathogenesis of CE is not completely understood, it is likely to involve the destruction of the arterial media, increased wall stress, thinning of the arterial wall and progressive dilatation of the coronary artery segment [7]. In addition, CE is also reported to be associated with increased plasma levels of inflammatory markers, cytokines and oxidative stress [8–10].

Oxidative stress represents an imbalance between the production of reactive oxygen species (ROS) and a biological system’s ability to detoxify the reactive intermediates and repair the resulting damage. It is thought to be involved in the development of many human diseases, including atherosclerosis [11,12]. Oxidative stress is usually associated with increased free radical formation and causes increased ROS concentrations. ROS modify proteins and phospholipids and lead to lipid peroxidation [13]. These substances may lead to inflammatory responses, such as leukocyte activation, the expression of adhesion molecules and the release of cytokines [14]. Atherosclerosis is the result of the oxidative modification of low density lipoproteins (LDL) in the arterial wall by ROS. ROS are key mediators of signaling pathways that underlie vascular inflammation in atherosclerosis starting from the development of a fatty streak, through lesion progression to ultimate plaque rupture [15].

Genomic DNA is also susceptible to being attacked by ROS, and multiple DNA repair enzymes protect DNA against such oxidative damage [16]. 8-Hydroxydeoxyguanine (8-OHdG) is a major component of the oxidative DNA damage induced by reactive free radicals and has been considered as a key biomarker of oxidative DNA damage in the literature [17,18]. On the contrary, base excision repair (BER) and nucleotide excision repair are the main DNA repair pathways that can help to remove 8-OHdG. Human 8-oxoguanine DNA glycosylase 1 (hOGG1) is a key enzyme of the base excision repair pathway and catalyzes the removal of 8-OHdG [19,20]. A functional polymorphism, Ser326Cys, is the common single nucleotide polymorphism (SNP) in the hOGG1 gene and is reported to be associated with risks of several cancers, diabetes and CAD [21–29]. Because CE is correlated to atherosclerosis and oxidative stress, we aimed to investigate the association between the functional hOGG1 Ser326Cys gene polymorphism and CE in a Chinese population in this study [6,30].

## 2. Results and Discussion

### 2.1. Clinical Characteristics

We finally enrolled 547 patients, including 53 (9.7%) patients with and 494 (90.3%) without CE. The average age is 61.8 ± 12.4 years old, and the percentage of males is 71.3%. Three-hundred seventy-four (68.4%) patients had significant CAD, including 120 (21.9%) with one-vessel disease (1VD), 96 (17.6%) with two-vessel disease (2VD) and 158 (28.9%) with three-vessel disease (3VD).

Table 1 summarizes the demographic information between those with and without CE. There was no significant difference between sex, age, incidence of diabetes, hypertension and hypercholesterolemia, history of smoking, family history of premature CAD, diseased vessels numbers, body mass index (BMI), history of previous acute coronary syndrome and the use of medications.

Table 2 summarizes the angiographic characteristics of the patients. Four-hundred ninety-four (90.3%) patients had no CE. Among 53 patients with CE, 42 (7.7%) patients had CE involving one major vessel (right coronary artery: 29 patients; left anterior descending artery: one patient; left circumflex artery: 12 patients); eight (1.4%) patients had CE involving two major vessels (five patients with right coronary artery and left anterior descending artery; three patients with right coronary artery and left circumflex artery); and, three (0.5%) patients had CE involving all three vessels. Furthermore, we did not find significant association between the number of ectatic vessels and hOGG1 Ser326Cys polymorphism (*p* = 0.165).

### 2.2. hOGG1 Ser326Cys Genotype

Among 547 study subjects, 192 (35.1%) were Cys/Cys, 279 (51.0%) Ser/Cys, and 76 (13.9%) were Ser/Ser genotype. The genotypic distribution was in Hardy–Weinberg equilibrium. Defining patients with hOGG1 Ser326Cys Cys/Cys genotype as the reference, the relative possibility of the presence of CE was 2.45 for patients with Ser/Cys genotype (odds ratio (OR): 2.45; 95% CI: 0.95–6.28; *p* = 0.063) and 2.53 for patients with Ser/Ser genotype (OR: 2.53; 95% CI: 1.22–5.25; *p* = 0.013) (Table 3). Using the dominant genetic model (Cys/Cys *vs.* Ser/Cys plus Ser/Ser), patients with the Ser allele were 2.51 times more likely to have CE than Cys/Cys genotype carriers (OR: 2.51; 95% CI: 1.23–5.11; *p* = 0.011).

Although there was no significant difference in the CAD number between non-CE and CE patients, we performed a subgroup analysis to avoid the possible influence of CAD on the hOGG1 Ser326Cys polymorphism. Among 547 study subjects, we excluded the patients with angiographic CAD in the non-CE patients to set up a control group without CAD (*n* = 156), and finally, 209 subjects were analyzed. Defining patients with hOGG1 Ser326Cys Cys/Cys genotype as the reference, the relative possibility of the presence of CE was 2.61 for patients with Ser/Cys genotype (OR: 2.61; 95% CI: 0.94–7.31; *p* = 0.067) and 2.80 for patients with Ser/Ser genotype (OR: 2.80; 95% CI: 1.28–6.13; *p* = 0.010) (Table 4). Using the dominant genetic model (Cys/Cys *vs.* Ser/Cys plus Ser/Ser), patients with the Ser allele were 2.76 times more likely to have CE than Cys/Cys genotype carriers (OR: 2.76; 95% CI: 1.29–5.90; *p* = 0.009).

Furthermore, we did not find a significant association between CAD and hOGG1 Ser326Cys polymorphism in our current study (*p* = 0.258).

### 2.3. Urinary Levels of 8OHdG

**Figure 1.**
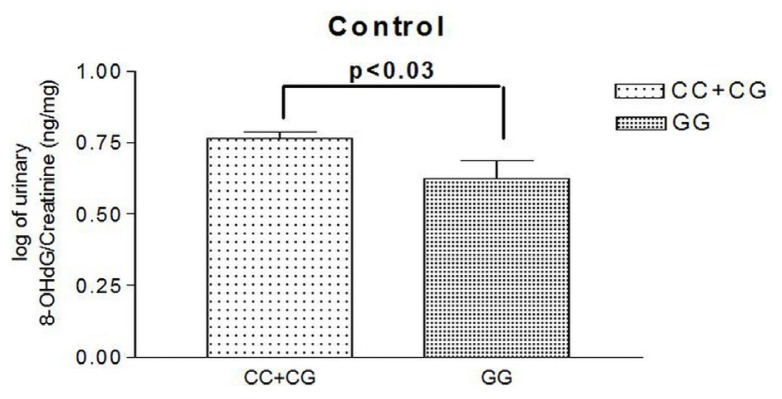
Urine 8-OHdG concentrations in different genetic variants. CC, Ser/Ser; CG, Ser/Cys; GG, Cys/Cys.

All of the urine samples were collected from healthy subjects during a health exam. We compared those carrying the hOGG1 Ser326Cys Ser genotype (*n* = 30) with those carrying the Cys/Cys genotype (*n* =15). Urine 8-OHdG levels were significantly higher in subjects carrying the hOGG1 Ser variant than in those with the Cys/Cys genotype (log of urine 8-OHdG/creatinine: 0.77 ± 0.126 *vs.* 0.66 ± 0.148 ng/mL; *p* = 0.022) (Figure 1).

### 2.4. Multivariate Regression Analysis

By stepwise binary logistic regression analysis after adjusting for age, sex, body mass index (BMI) and traditional coronary risk factors, including hypertension, diabetes, hypercholesterolemia, smoking and family history of premature CAD, hOGG1 Ser326Cys polymorphism in the dominant model and BMI were significantly independent predictors for the presence of CE (*p* = 0.011 and 0.009, respectively) (Table 5).

### 2.5. Discussion

There were two major findings in the present study. First, subjects carrying the hOGG1 Ser326Cys Ser variant had 1.55 times the risk for having coronary ectasia. Second, hOGG1 Ser326Cys polymorphism in the dominant model is associated with the presence of coronary ectasia after multivariate logistic regression analysis.

The hOGG1 repair enzyme is an important component responsible for repairing the 8-OHdG, which is a sensitive biomarker for indicating the presence of oxidative DNA damage [19,20]. 8-OHdG is excreted in to the urine during DNA repair. Urinary 8-OHdG has been measured frequently to indicate the extent of oxidative damage, due to its simple and noninvasive technique. Numerous pieces of evidence have indicated that urinary 8-OHdG not only is a biomarker of generalized, cellular oxidative stress, but might also be a risk factor for cancer, atherosclerosis and diabetes [18,30–33]. In addition, there were many studies focusing on the functional hOGG1 Ser326Cys polymorphism [21–29,34,35]. Aka *et al.* reported that the DNA repair capacity in individuals with Ser/Cys or Cys/Cys OGG1 genotypes was lower than those with the Ser/Ser hOGG1 genotype [34]. Thomas D. *et al.* found that the inactivation of hOGG1 increases the frequency of G–C to T–A transversion mutations in Saccharomyces cerevisiae [35]. These studies revealed that the hOGG1 Cys326 variant causes the reduction of repair enzyme activity to remove 8-OHdG and leads to the accumulation of 8-OHdG and increases DNA damage [12].

CAD is characterized by the formation of atherosclerotic plaque within the walls of the coronary arteries and is also a chronic inflammatory disease [36]. The pathogenesis of atherosclerosis is related to oxidative stress, which causes the oxidative modification of LDL by ROS and further leads to atherosclerotic plaques progression and possible rupture [11,12,15]. Our recent study also revealed that hOGG1 Ser326Cys polymorphism was associated with the risk of CAD and multi-vessel disease and might be a novel genetic marker for the development and severity of CAD in the Chinese population [29].

CE is a variant of coronary artery abnormality [6]. It may be congenital or acquired. Acquired causes include CAD, various inflammatory and infectious diseases and Kawasaki disease [2–6,8–10]. However, most cases of CE are correlated with atherosclerosis and had the coexistence of obstructive CAD [2,6]. In addition, CE was also reported to be associated with oxidative stress in a recent study [10]. Sezen Y. *et al.* found that total oxidant status and the oxidative stress index were significantly increased, and the total antioxidant status was decreased in the CE group compared to the control group [10]. In the current study, we discovered that CE is significantly associated with hOGG1 Ser326Cys gene polymorphism in a dominant model after adjusting the possible confounding factors. This finding was still noted in the subgroup analysis using patients without CAD as the control group. Although CE is considered as a variant of CAD, its pathogenesis has not yet been completely illustrated. Even in patients with isolated CE without coronary stenosis, there is still a higher incidence of adverse events in this population compared to people with normal coronary arteries. CE could produce sluggish blood flow and predisposes patients to acute myocardial infarction, even without obstructed coronary arteries [37]. In the current study, we did not find a significant association between CAD and hOGG1 Ser326Cys polymorphism, which might be related to a limited sample size in comparison with our previous study. However, we discovered a significant association between CE and hOGG1 Ser326Cys polymorphism. These results might suggest that patients with CE have greater oxidative stress than those with CAD. In addition, we also found that urinary 8-OHdG levels were significantly higher in subjects carrying the hOGG1 Ser variant than in those with the Cys/Cys genotype. These findings could further confirm the association of oxidative stress with CE and indicates that the hOGG1 Ser326Cys gene variant might play a role in the individual’s susceptibility to the development of CE in the Chinese population.

A previous study had reported that obesity was the only coronary risk factor that showed a statistically significant correlation with CE [38]. In this study, obesity was present in 60% of the patients who had CE, compared with 42% of patients who did not have CE. This finding is compatible with the findings in our study. In our study, higher BMI was still significantly associated with CE after multivariate regression analysis. Hence, obesity might also be a risk factor in the development of CE. The possible mechanism might be related to the interaction of connective tissue elements with lipoprotein particles, which weakens the structure of the arterial wall and the association between obesity and abnormal lipoprotein metabolism [38]. However, further studies are needed to clarify the detailed mechanism.

There are several limitations of this study. First, the findings should be taken with caution, because they are based on a limited sample size. Therefore, the results need to be replicated for confirmation by further studies using larger samples. Second, the association between hOGG1 Ser326Cys genotypes and urinary 8-OHdG levels were based on limited samples from healthy subjects instead of all enrolled patients. However, our data might reduce the confounding factors and truly demonstrate the influence of the genetic variant on urine 8-OHdG concentrations. Third, since this is only a clinical association study, a detailed discussion on the potential mechanisms should be limited.

## 3. Experimental Section

### 3.1. Study Subjects

We consecutively evaluated patients scheduled for diagnostic coronary angiography from Kaohsiung Medical University Hospital (KMUH) in Taiwan. Patients with a history of coronary artery bypass surgery, a history of percutaneous coronary intervention, uninterpretable restriction digest results or inadequate angiograms for evaluation were excluded. We collected patients’ demographic and baseline information, including sex, age, duration of chest pain, history of diabetes, hypertension, hypercholesterolemia, cigarette smoking and medication. The research protocol was approved and registered by the local institution ethics committee (KMUH-IRB), and informed consent was obtained from the patients.

### 3.2. Coronary Angiography

The coronary artery angiography films were reviewed by two experienced cardiologists blind to the patients’ clinical characteristics. A third reviewer blinded to the readings of the first two reviewers served as the arbitrator of the differences, if needed. Coronary angiography was performed by the femoral or radial approach with 6 French diagnostic catheters. Images were recorded in multiple projections for left and right coronary arteries. Coronary artery stenosis was determined by quantitative coronary angiography. The presence of CAD is defined as coronary diameter stenosis more than 50%. CE is defined as the diameter of the ectatic segment being more than 1.5 times larger compared with an adjacent healthy reference segment [2]. The recorded data also included the location of CAD and CE and the number and percentage of stenosis of the diseased vessels.

### 3.3. DNA Isolation and Genotyping

Blood samples for genotyping (hOGG1 polymorphisms in codon 326, rs1052133) were collected during coronary catheterization. Genomic DNA was extracted from EDTA whole blood samples following standard procedures and stored at −20 ºC. The genotype was determined with the TaqMan allelic discrimination assay (Applied Biosystems, ABI, Foster City, CA, USA), following the manufacturer’s instructions. SNP allele-specific primers and probes were designed and synthesized by ABI (Foster City, CA, USA). Nucleotide primers were 5′CTCCTACAGGTGCTGTTCAGT3′ (forward primer) and 5′ACCCTTTCTGCGCTTTGCT3′ (reverse primer). The probe for Ser326 was 5′CCAATCCCGCCATGC3′, and for Cys326, it was 5′CAATGCCGCCATGC3′. Probe 5′ ends were labeled, the Cys326 probe with 6-carboxy-fluorescein (FAM) and the Ser326 with 2′-chloro-7′-phenyl-1,4-dichloro-6-carboxyfluorescein (VIC). After thermal cycling (50 ºC for 2 min, 95 ºC for 10 min, then 40 cycles at 95 ºC for 15 s and at 60 ºC for 90 s), allelic discrimination was performed (Applied Biosystems 7900 real-time PCR system, ABI, Foster City, CA, USA).

### 3.4. Urine 8OHdG Measurement by Enzyme-Linked Immunosorbent Assay (ELISA)

The urine values of 8-OHdG could be determined in 45 subjects from a healthy general population (15 subjects for each genotype). Urine samples were stored in polypropylene tubes or polystyrene tubes at −80 ºC until the time of analysis. To determine urine 8-OHdG levels, an 8-OHdG Check ELISA kit (from Japan Institute for the Control of Aging, Fukuroi, Japan) was used. The kits utilize the highly specific monoclonal 8-OHdG antibody (clone N45.1). All assays were done according to the manufacturer’s instructions.

### 3.5. Statistical Analysis

All data were expressed as the mean ± standard deviation. If the minor allele frequency (MAF) for hOGG1 Ser326Cys polymorphism among the non-CE group was estimated to be 0.384, the study should enroll at least 51 patients with the non-CE group to have more than 80% statistical power to detect an association with Odds ratios (ORs) ≥ 2.5. The independent *t-*test was used for continuous variables analysis between two groups. The genotypic distribution was tested by the Hardy–Weinberg equilibrium. A chi-square test was used to compare categorical data. ORs with 95% confidence intervals (CI) were calculated as a measure of the association of hOGG1 genotypes in a different genetic analysis model with CE. The relevant correlated variables and the appropriate genetic model were further analyzed by logistic regression analysis. All *p*-values were two-sided with a significant level of *p* < 0.05. The Statistical Package for the Social Sciences (SPSS) 11.0 for Windows (SPSS Inc., Chicago, IL, USA) was used for statistical analysis.

## 4. Conclusions

To our best knowledge, this is the first report discussing the association between Ser326Cys gene polymorphism and CE. In this study, we discovered a significant association of the hOGG1 Ser326Cys gene variant in the dominant model with CE development. These findings indicate that the hOGG1 Ser326Cys gene variant might be a novel genetic marker in the susceptibility to the development of CE in the Chinese population.

## Figures and Tables

**Table 1.** Patients’ baseline characteristics.

Parameters	Non-CE *N* = 494	CE *N* = 53	*p*-value

	*N* (%) or Mean ± SD		
Sex (male)	348 (70.4)	42 (79.2)	0.178
Age (years)	61.6 ± 12.4	62.8 ± 12.7	0.502
DM	180 (36.4)	15 (28.3)	0.240
Hypertension	315 (63.8)	40 (75.5)	0.090
Smoking	257 (52.0)	32 (60.4)	0.247
Family history of premature CAD	11 (2.2)	1 (1.9)	1.000
Hypercholesterolemia (%)	255 (51.6)	21 (39.6)	0.097

CAD number			0.431
Non-significant lesion	156 (31.7)	15 (28.3)	
1-vessel disease	107 (21.7)	13 (24.5)	
2-vessel disease	83 (16.9)	13 (24.5)	
3-vessel disease	146 (29.7)	12 (22.6)	
BMI	25.3 ± 3.5	26.6 ± 5.0	0.074
Previous ACS	30 (9.4)	23 (10.1)	0.768

Medication			
Aspirin	210 (42.5)	25 (47.2)	0.515
Clopidogrel	48 (9.7)	7 (13.2)	0.422
β-blocker	177 (35.8)	20 (37.7)	0.784
Nitrate	143 (28.9)	17 (32.1)	0.634
ACEI	99 (20.0)	11 (20.8)	0.902
ARB	101 (20.4)	11 (20.8)	0.958
CCB	138 (27.9)	13 (24.5)	0.598
Diuretic	79 (16.0)	7 (13.2)	0.597
Statin	98 (19.8)	9 (17.0)	0.618

Abbreviations: SD, standard deviation; CAD, coronary artery disease; CE, coronary ectasia; DM, diabetes mellitus; ACS, acute coronary syndrome; BMI, body mass index; ACEI, angiotensin converting enzyme inhibitor; ARB, angiotensin receptor blocker; CCB, calcium channel blocker.

**Table 2.** Angiographic characteristics among the 53 coronary ectasia patients.

Ectasia location	*N* (%)
Right coronary artery	29 (5.3)
Left anterior descending artery	1 (0.2)
Left circumflex artery	12 (2.2)
Right coronary artery + left anterior descending artery	5 (0.9)
Right coronary artery + left circumflex artery	3 (0.5)
Left anterior descending artery + left circumflex artery	0
All three vessels	3 (0.5)

Number of ectatic vessels in patients with coronary ectasia	
1	42 (7.7)
2	8 (1.4)
3	3 (0.5)

**Table 3.** hOGG1 Ser326Cys genotype and ectasia status.

Polymorphism site	Non-CE *N* (%)	CE *N* (%)	OR (95% CI)	*p*-value	aOR * (95% CI)	*p*-value
**Allele frequency**						
G	609 (61.6)	54 (50.9)	1		1	
C	379 (38.4)	52 (49.1)	1.55 (1.04–2.31)	0.033	1.55 (1.03–2.33)	0.037
**Genotype**						
GG	182 (36.8)	10 (18.9)	1		1	
CG	245 (49.6)	34 (64.2)	2.45 (0.95–6.28)	0.063	2.41 (0.92–6.29)	0.074
CC	67 (13.6)	9 (17.0)	2.53 (1.22–5.25)	0.013	2.624 (1.24–5.53)	0.011
**Dominant model**						
GG	182 (36.8)	10 (18.9)	1		1	
CG + CC	312 (63.2)	43 (81.1)	2.51 (1.23–5.11)	0.011	2.57 (1.25–5.32)	0.011

aOR, adjusted odds ratio; CE, coronary ectasia; CI, confidence interval; hOGG1, Human 8-oxoguanine DNA glycosylase; OR: odds ratio.

* aOR is based on logistic regression adjusted for age, sex, body mass index and traditional coronary risk factors, including hypertension, diabetes, hypercholesterolemia, smoking and family history of premature coronary artery disease.

**Table 4.** hOGG1 Ser326Cys genotype and ectasia status in subgroup patients.

Polymorphism site	Control group *N* (%)	CE group *N* (%)	OR (95% CI)	*p*-value	aOR * (95% CI)	*p*-value
**Allele frequency**						
G	196 (62.8)	54 (50.9)	1		1	
C	116 (37.2)	52 (49.1)	1.63 (1.04–2.54)	0.032	1.64 (1.00–2.68)	0.049
**Genotype**						
GG	61 (39.1)	10 (18.9)	1		1	
CG	74 (47.4)	34 (64.2)	2.61 (0.94–7.31)	0.067	2.65 (0.84–8.34)	0.097
CC	21 (13.5)	9 (17.0)	2.80 (1.28–6.13)	0.010	2.941 (1.24–7.00)	0.015
**Dominant model**						
GG	61 (39.1)	10 (18.9)	1		1	
CG + CC	95 (60.9)	43 (81.1)	2.76 (1.29–5.90)	0.009	2.88 (1.24–6.67)	0.014

aOR, adjusted odds ratio; CE, coronary ectasia; CI, confidence interval; hOGG1, Human 8-oxoguanine DNA glycosylase; OR: odds ratio.

* aOR is based on logistic regression adjusted for age, sex, body mass index and traditional coronary risk factors, including hypertension, diabetes, hypercholesterolemia, smoking and family history of premature coronary artery disease.

**Table 5.** Results obtained from the binary logistic analysis.

Variables	OR	95% CI	*p*-value
Age	1.02	0.99–1.05	0.132
Sex (male *vs*. female)	1.57	0.64–3.81	0.325
Hypertension	1.86	0.94–3.71	0.077
Diabetes	0.53	0.27–1.01	0.055
Hypercholesterolemia	0.56	0.31–1.03	0.062
Body mass index	1.11	1.03–1.20	0.009
Smoking	0.88	0.42–1.85	0.736
Family history of premature CAD	1.27	0.15–10.59	0.826
hOGG1 CG + CC *vs.* GG	2.57	1.25–5.32	0.011

CAD, coronary artery disease; CI, confidence interval; hOGG1, Human 8-oxoguanine DNA glycosylase; OR, odds ratio.

## References

[1] Oliveros R.A., Falsetti H.L., Carroll R.J., Heinle R.A., Ryan G.F. (1974). Atherosclerotic coronary artery aneurysm: Report of five cases and a review of the literature. Arch. Intern. Med.

[2] Swaye P.S., Fisher L.D., Litwin P., Vignola P.A., Judkins M.P., Kemp H.G., Mudd J.G., Gosselin A.J. (1983). Aneurysmal coronary artery disease. Circulation.

[3] Hartnell G.G., Parnell B.M., Pridie R.B. (1985). Coronary artery ectasia. Its prevalence and clinical significance in 4993 patients. Br. Heart. J.

[4] Yilmaz H., Sayar N., Yilmaz M., Tangürek B., Cakmak N., Gürkan U., Gül M., Simşek D., Bolca O. (2008). Coronary artery ectasia: clinical and angiographical evaluation. Turk. Kardiyol. Dern. Ars.

[5] Sharma S.N., Kaul U., Sharma S., Wasir H.S., Manchanda S.C., Bahl V.K., Talwar K.K., Rajani M., Bhatia M.L. (1990). Coronary arteriographic profile in young and old Indian patients with ischaemic heart disease: A comparative study. Indian Heart J.

[6] Swanton R.H., Thomas M.L., Coltart D.J., Jenkins B.S., Webb-Peploe M.M., Williams B.T. (1978). Coronary artery ectasia—A variant of occlusive coronary arteriosclerosis. Br. Heart. J.

[7] Rodbars S., Ikeda K., Montes M. (1967). An analysis of mechanisms of poststenotic dilatation. Angiology.

[8] Ozbay Y., Akbulut M., Balin M., Kayancicek H., Baydas A., Korkmaz H. (2007). The level of hs-CRP in coronary artery ectasia and its response to statin and angiotensin-converting enzyme inhibitor treatment. Mediators Inflamm.

[9] Li J.J., Nie S.P., Qian X.W., Zeng H.S., Zhang C.Y. (2009). Chronic inflammatory status in patients with coronary artery ectasia. Cytokine.

[10] Sezen Y., Bas M., Polat M., Yildiz A., Buyukhatipoglu H., Kucukdurmaz Z., Kaya Z., Demirbag R. (2010). The relationship between oxidative stress and coronary artery ectasia. Cardiol. J.

[11] Singh U., Jialal I. (2006). Oxidative stress and atherosclerosis. Pathophysiology.

[12] Vogiatzi G., Tousoulis D., Stefanadis C. (2009). The role of oxidative stress in atherosclerosis. Hellenic. J. Cardiol.

[13] Suzuki S., Kaneko M., Chapman D.C., Dhalla N.S. (1991). Alterations in cardiac contractile proteins due to oxygen free radicals. Biochim. Biophys. Acta.

[14] Biglioli P., Cannata A., Alamanni F., Naliato M., Porqueddu M., Zanobini M., Tremoli E., Parolari A. (2003). Biological effects of off-pump *vs.* on-pump coronary artery surgery: Focus on inflammation, hemostasis and oxidative stress. Eur. J. Cardiothorac. Surg.

[15] Madamanchi N.R., Hakim Z.S., Runge M.S. (2005). Oxidative stress in atherogenesis and arterial thrombosis: The disconnect between cellular studies and clinical outcomes. J. Thromb. Haemost.

[16] Wiseman H., Halliwell B. (1996). Damage to DNA by reactive oxygen and nitrogen species: Role in inflammatory disease and progression to cancer. Biochem. J.

[17] Shibutani S., Takeshita M., Grollman A.P. (1991). Insertion of specific bases during DNA synthesis past the oxidation-damaged base 8-oxodG. Nature.

[18] Wu L.L., Chiou C.C., Chang P.Y., Wu J.T. (2004). Urinary 8-OHdG: A marker of oxidative stress to DNA and a risk factor for cancer, atherosclerosis and diabetics. Clin. Chim. Acta.

[19] Croteau D.L., Bohr V.A. (1997). Repair of oxidative damage to nuclear and mitochondrial DNA in mammalian cells. J. Biol. Chem.

[20] Bruner S.D., Norman D.P., Verdine G.L. (2000). Structural basis for rec ognition and repair of the endogenous mutagen 8-oxoguanine in DNA. Nature.

[21] Li H., Hao X., Zhang W., Wei Q., Chen K. (2008). The hOGG1 Ser326Cys polymorphism and lung cancer risk: a meta-analysis. Cancer. Epidemiol. Biomarkers Prev.

[22] Xing D.Y., Tan W., Song N., Lin D.X. (2001). Ser326Cys polymorphism in hOGG1 gene and risk of esophageal cancer in a Chinese population. Int. J. Cancer.

[23] Xu J., Zheng S.L., Turner A., Isaacs S.D., Wiley K.E., Hawkins G.A., Chang B.L., Bleecker E.R., Walsh P.C., Meyers D.A. (2002). Associations between hOGG1 sequence variants and prostate cancer susceptibility. Cancer Res.

[24] Tsukino H., Hanaoka T., Otani T., Iwasaki M., Kobayashi M., Hara M., Natsukawa S., Shaura K., Koizumi Y., Kasuga Y. (2004). hOGG1 Ser326Cys polymorphism, interaction with environmental exposures, and gastric cancer risk in Japanese populations. Cancer. Sci.

[25] Jiao X., Huang J., Wu S., Lv M., Hu Y., Jianfu, Su X., Luo C., Ce B. (2007). hOGG1 Ser326Cys polymorphism and susceptibility to gallbladder cancer in a Chinese population. Int. J. Cancer.

[26] Zhao H., Qin C., Yan F., Wu B., Cao Q., Wang M., Zhang Z., Yin C. (2011). hOGG1 Ser326Cys polymorphism and renal cell carcinoma risk in a Chinese population. DNA. Cell. Biol.

[27] Wang C.L., Hsieh M.C., Hsin S.C., Lin H.Y., Lin K.D., Lo C.S., Chen Z.H., Shin S.J. (2006). The hOGG1 Ser326Cys gene polymorphism is associated with decreased insulin sensitivity in subjects with normal glucose tolerance. J. Hum. Genet.

[28] Sun C., Liu X., Zhang H., Guo W., Cai Z., Chen H., Zhang K., Zhu D., Wang Y. (2010). Functional polymorphism of hOGG1 gene is associated with type 2 diabetes mellitus in Chinese population. Mol. Cell. Endocrinol.

[29] Wang C.L., Lin T.H., Lin H.Y., Sheu S.H., Yu M.L., Hsiao P.J., Lin K.D., Hsu C., Yang Y.H., Shin S.J. (2010). The 8-oxoguanine glycosylase I (hOGG1) Ser326Cys variant affects the susceptibility to multi-vessel disease in Taiwan coronary artery disease patients. Thromb. Res.

[30] Chiou C.C., Chang P.Y., Chan E.C., Wu T.L., Tsao K.C., Wu J.T. (2003). Urinary 8-hydroxydeoxyguanosine and its analogs as DNA marker of oxidative stress: Development of an ELISA and measurement in both bladder and prostate cancers. Clin. Chim. Acta.

[31] Negishi H., Ikeda K., Kuga S., Noguchi T., Kanda T., Njelekela M., Liu L., Miki T., Nara Y., Sato T. (2001). The relation of oxidative DNA damage to hypertension and other cardiovascular risk factors in Tanzania. J. Hypertens.

[32] Serdar M., Sertoglu E., Uyanik M., Tapan S., Akin K., Bilgi C., Kurt I. (2012). Comparison of 8-hydroxy-2′-deoxyguanosine (8-OHdG) levels using mass spectrometer and urine albumin creatinine ratio as a predictor of development of diabetic nephropathy. Free. Radic. Res.

[33] Xu G.W., Yao Q.H., Weng Q.F., Su B.L., Zhang X., Xiong J.H. (2004). Study of urinary 8-hydroxydeoxyguanosine as a biomarker of oxidative DNA damage in diabetic nephropathy patients. J. Pharm. Biomed. Anal.

[34] Aka P., Mateuca R., Buchet J.P., Thierens H., Kirsch-Volders M. (2004). Are genetic polymorphisms in OGG1, XRCC1 and XRCC3 genes predictive for the DNA strand break repair phenotype and genotoxicity in workers exposed to low dose ionising radiations?. Mutat. Res.

[35] Thomas D., Scot A.D., Barbey R., Padula M., Boiteux S. (1997). Inactivation of OGG1 increases the incidence of G. C-->T. A transversions in Saccharomyces cerevisiae: Evidence for endogenous oxidative damage to DNA in eukaryotic cells. Mol. Gen. Genet.

[36] Ross R. (1999). Atherosclerosis--an inflammatory disease. N. Engl. J. Med.

[37] Befeler B., Aranda M.J., Embi A., Mullin F.L., El-Sherif N., Lazzara R. (1977). Coronary artery aneurysms: study of their etiology, clinical course and effect on left ventricular function and prognosis. Am. J. Cardiol.

[38] Waly H.M., Elayda M.A., Lee V.V., El-Said G., Reul G.J., Hall R.J. (1997). Coronary artery ectasia in Egyptian patients with coronary artery disease. Tex. Heart. Inst. J.

